# A rabbit model for assessing symblepharon after alkali burn of the superior conjunctival sac

**DOI:** 10.1038/s41598-019-50286-x

**Published:** 2019-09-25

**Authors:** Yanwei Kang, Shaowei Li, Chang Liu, Mintian Liu, Shuai Shi, Man Xu, Jingliang He, Tao Zhang

**Affiliations:** 1 Aier School of Ophthalmology, Central South University, Changsha, Hunan China; 2 Aier Institute of Cornea, Beijing, China; 3 Department of Ophthalmology, Beijing Aier Intech Eye Hospital, Beijing, China; 4 Department of Ophthalmology, Binzhou Aier Eye Hospital, Binzhou, Shandong China

**Keywords:** Eye abnormalities, Trauma

## Abstract

Symblepharon due to chemical burns affects ocular surface health, and there are currently no satisfactory treatments. To improve our understanding of symblepharon, an appropriate animal model is urgently needed. We established a rabbit model of superior conjunctival sac alkaline burn to evaluate symblepharon severity. Alkali burns were induced in rabbits by contacting the superior conjunctival sac with 2 N NaOH-soaked semicircle filter paper (10 mm diameter) for 60 s, 90 s or 120 s. Clinical and histological features were examined, symblepharon severity was evaluated via conjunctival sac depth (grade I - IV) and volume measurements (grade a-d) post-injury at 4 weeks. With increasing alkali burn duration, corneal perforation and symblepharon severity increased. The 60 s group manifested a sub-conjunctiva scar. The 90 s group featured localized adhesion. The 120 s group was characterized by extensive scar hyperplasia and adhesion. The rabbit model exhibited stable and reliable symblepharon following an alkali burn of the superior conjunctival sac. For further research, 90 s is a suitable duration for conjunctival sac burn. The volume measured using conjunctival sac casting was considered when developing a successful evaluation system for symblepharon severity.

## Introduction

Symblepharon is one of the most challenging complications at the late stage of alkali burn. The deleterious effects of symblepharon on the ocular surface include dry eye, inadequate blinking, eyelid malposition, mechanical extraocular movement restriction and additional abnormal appearance. Various approaches have been developed for the treatment of symblepharon. The use of systemic steroids^1^, immunosuppression^2,3^, or stronger agents can help mitigate ocular surface inflammation, which drives the cicatricial process. Tissue substitutes such as conjunctival graft^3,4^, amniotic membrane^5,6^, oral mucosa^7^, and nasal mucosa^8^ have been used to cover the exposed surfaces after symblepharon lysis. Adjunctive measures have been evaluated to prevent potential re-adhesion, such as symblepharon rings, silicone sheets^9^, mitomycin C (MMC)^10^, anchoring sutures^11^, bevacizumab^12^, and beta irradiation^1,13^.

Despite the efforts described above, the formation and recurrence of symblepharon after alkali burn remain highly variable among centers^6,10,14–16^. To obtain a better understanding of symblepharon, a suitable animal model is needed. Current animal models for alkali burns focus on the role of alkali damage to the cornea. A conventional study used round filter paper discs immersed in NaOH to contact the central axis of the cornea to induce alkali wounds^17–19^. Zhou *et al*.^20^ placed a circular cotton sponge soaked in NaOH onto the central cornea to initiate alkali burns. Haddox *et al*.^21^ induced alkali burns by applying NaOH solution directly to the surface of central corneas. These studies, however, did not mention the accompanied symblepharon. By the time the cornea was burned, extensive conjunctiva damage had already been caused, and symblepharon may have occurred later. Herein, we report our experimental findings modelling symblepharon in rabbits using alkaline conditions to destroy conjunctiva other than the cornea. This study provides a laboratory and clinical research basis for the prevention and treatment of alkali-injured symblepharon.

## Results

### Clinical observation

**Figure 1:**
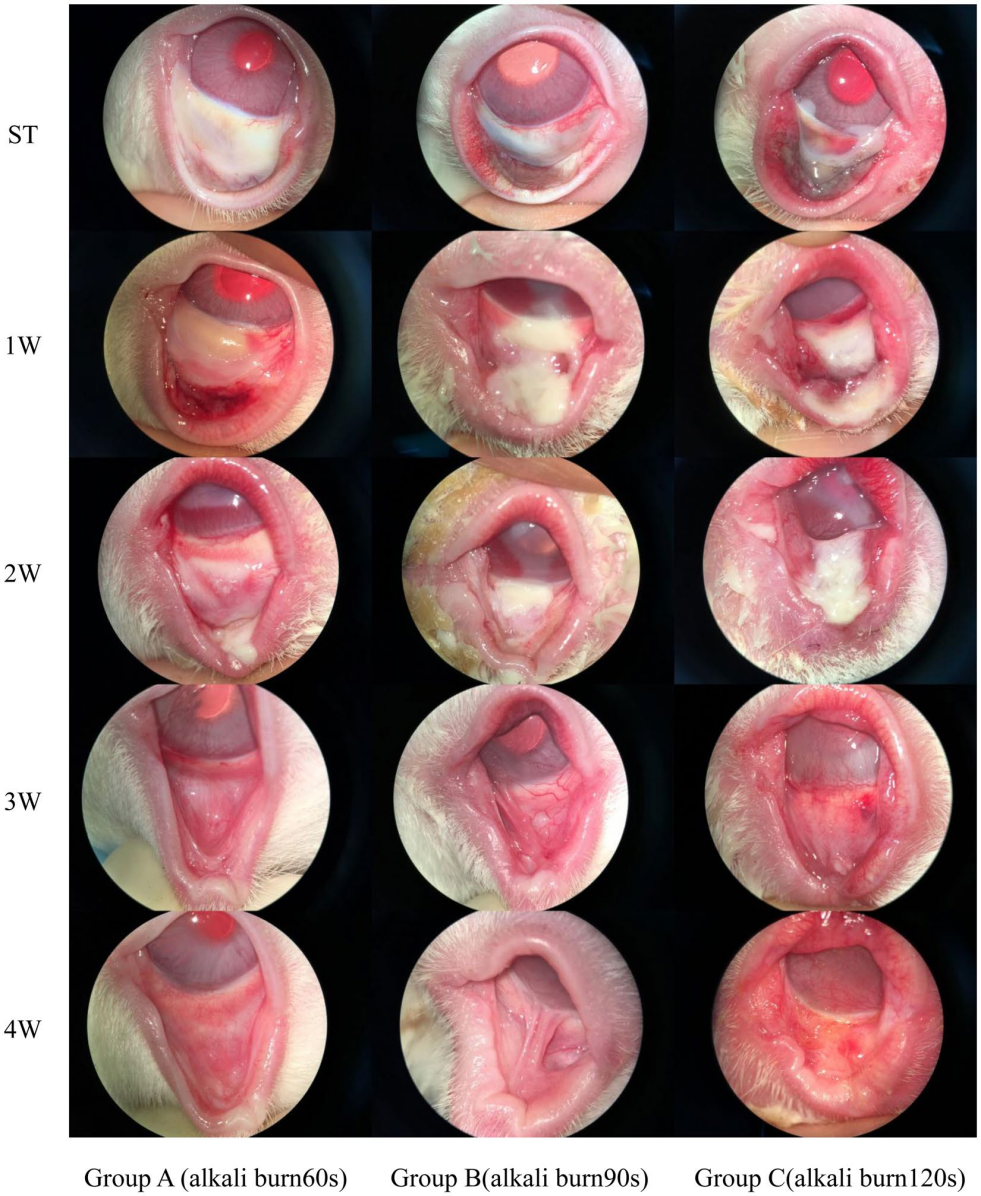
Alkali-burned superior conjunctival sac following 4 weeks slit lamp microscopy examination in group A (left), group B (middle) and group C (right).

The clinical manifestations at different stages after superior conjunctival sac alkali burn are shown in Table 1 and Fig. 1.

**Table 1:** Alkali-burned superior conjunctival sac following 4 weeks slit lamp microscopy examination.

Time post-injury	Group A (alkali burn60s)	Group B(alkali burn90s)	Group C(alkali burn120s)
ST	extensive chemosis, conjunctival ischemia appear colorless and transparent, mild corneal edema	extensive conjunctival ischemia, choroid were mostly seen through the burn area, limbus ischemia and palpebral edge hyperemia near the burn area	total conjunctival ischemic necrosis turn white, black barsus necrosis and white corneal opacity surrounding burn area, corneal edema
1 W	chemosis, palpebral subconjunctival hemorrhages, a little limbus vessel dilation	conjunctival necrosis and secretions, limbal hyperemia	conjunctiva and lid margin necrosis,corneal opacity, limbus angiogenesis
2 W	small necrosis area in burn area, corneal angiogenesis	ulcer induced by conjunctival necrosis exfoliated, corneal angiogenesis	conjunctiva and lid margin necrosis and secretions, corneal opacity, corneal neovascularization
3 W	conjunctival scar, a little corneal pannus	connective tissue fiber band between conjunctival, some limbal vascularized area	hypertrophic burn scars in conjunctival and eyelid margin, eyelid margin defect, corneal pannus
4 W	a little connective tissue fiber band and corneal pannus	obvious adhesion between bulbar conjunctiva and palpebral conjunctiva, corneal pannus, angular deformity of eyelid margin in an “M-shaped”	extensive hyperplastic scar and adhesion, corneal opacity, corneal pannus, eyelid margin defect, angular deformity of eyelid margin was “volcano-like”

### Superior conjunctival fornix depth

Compared with preoperative values, the conjunctival sac depth was decreased on postoperative week 4 in each group **(**Table 2**)**. For the distance from the limbus to the fornix, the baseline centre locations in group A, group B and group C were13.63 ± 0.98 mm, 13.58 ± 0.87 mm and 13.58 ± 0.90 mm, respectively. At week 4, these values had reduced to10.54 ± 1.37 mm, 6.04 ± 2.09 mm and 1.83 ± 1.70 mm, respectively. For the distance from the fornix to the lid margin, the preoperative centre depths were 20.75 ± 0.97 mm in group A, 20.50 ± 1.45 mm in group B and 19.83 ± 1.03 mm in group C. Four weeks after burn, the centre depths were 15.25 ± 3.74 mm, 8.08 ± 3.58 mm and 4.83 ± 2.51 mm, respectively, in group A, group B and group C. The differences in the reduction in this distance were statistically significant among the three groups (*P* = 0.000). The temporal and nasal depths were also significantly decreased to different degrees among the three groups (*P* = 0.000). Regarding the different positions among groups, the change from the fornix to the eyelid margin was greater in group B at the centre and negligible in groups A and C at all locations (*P* = 0.000), the change from the fornix to the limbus was significantly greater at all positions in group A and at the centre in both groups B and C (*P* = 0.000), whereas the change was negligible between the temporal and nasal region in both groups B and C.

**Table 2:** The superior conjunctival sac depth (mm).

Superior conjunctival sac depth(mm)		Group A					Group B					Group C					Decrement among groups (*P*-value)
		(n = 12)					(n = 12)					(n = 12)					
		Pre-operative	Post-operative	Δdepth	Decrement (%)	pre VS post (*P*-value)	Pre-operative	Post-operative	Δdepth	Decrement (%)	pre VS post (*P*-value)	Pre-operative	Post-operative	Δdepth	Decrement (%)	Pre VS post (*P*-value)	
Limbus -fornix	Temporal	13.00 ± 0.93	11.67 ± 1.48	1.33 ± 0.65	0.11 ± 0.05	0.000	13.04 ± 0.89	7.58 ± 2.03	5.46 ± 1.41	0.42 ± 0.13	0.000	13.25 ± 1.22	2.46 ± 2.02	10.79 ± 1.30	0.82 ± 0.14	0.000	0.000
		(12–15)	(10–14.5)	(0–2)	(0–16.67)		(12–14.5)	(4–10)	(4–8)	(28.57–66.67)		(12–15)	(0–6)	(8–12)	(60–100)		
	Centre	13.63 ± 0.98	10.54 ± 1.37	3.08 ± 0.51	0.23 ± 0.05	0.000	13.58 ± 0.87	6.04 ± 2.09	7.54 ± 1.39	0.56 ± 0.14	0.000	13.58 ± 0.90	1.83 ± 1.70	11.75 ± 0.97	0.87 ± 0.12	0.000	0.000
		(12–15)	(8–13)	(2–4)	(13.33–33.33)		(12–15)	(2–8)	(6–10.5)	(42.86–83.33)		(12–15)	(0–5)	(10–13)	(66.67–100)		
	Nasal	12.25 ± 1.12	10.08 ± 1.58	2.17 ± 0.78	0.18 ± 0.07	0.000	12.42 ± 1.14	7.13 ± 1.91	5.29 ± 1.16	0.43 ± 0.12	0.000	12.33 ± 1.37	1.71 ± 1.51	10.63 ± 0.64	0.87 ± 0.11	0.000	0.000
		(11–14)	(8–14)	(0–3)	(0–27.27)		(11–14)	(4–10)	(4–7)	(28.57–63.64)		(10–14)	(0–5)	(9–11)	(64.29–100)		
Decrement among positions (*P*-value)		0.000					0.000					0.021					
Fornix-lid margin	Temporal	20.58 ± 1.24	16.25 ± 3.43	4.33 ± 2.74	0.21 ± 0.15	0.000	19.83 ± 1.40	9.67 ± 3.24	10.17 ± 2.00	0.52 ± 0.13	0.000	19.42 ± 1.31	5.29 ± 2.31	14.13 ± 1.30	0.73 ± 0.11	0.000	0.000
		(19–23)	(9–18.5)	(2–10)	(10–52.63)		(17–22)	(5–16)	(6–13.5)	(27.27–71.05)		(17–22)	(0–8)	(12.5–17)	(61.9–100)		
	Ccentral	20.75 ± 0.97	15.25 ± 3.74	5.50 ± 3.06	0.27 ± 0.16	0.000	20.50 ± 1.45	8.08 ± 3.58	12.42 ± 2.50	0.61 ± 0.15	0.000	19.83 ± 1.03	4.83 ± 2.51	15.00 ± 1.77	0.76 ± 0.12	0.000	0.000
		(19–22)	(7–18)	(4–12)	(18.18–63.16)		(18–22)	(3–16)	(6–13.5)	(27.27–83.33)		(19–22)	(0–8)	(13–19)	(61.9–100)		
	Nasal	19.67 ± 1.23	15.08 ± 3.40	4.58 ± 2.57	0.24 ± 0.15	0.000	18.75 ± 1.66	8.71 ± 3.22	10.04 ± 1.86	0.55 ± 0.14	0.000	18.67 ± 0.89	4.25 ± 2.30	14.42 ± 1.51	0.78 ± 0.11	0.000	0.000
		(18–22)	(8–18)	(3–10)	(15–55.56)		(16–21)	(4–15)	(6–12.5)	(28.57–75)		(17–20)	(0–8)	(12–17)	(60–100)		
Decrement among positions (*P*-value)		0.567					0.016					0.376					

The differences in the reduction were statistically significant among the three groups (*P* = 0.000). In group A, there were a statistically significant differences in the change from fornix to limbus among all the positions (*P* = 0.000), there were no differences in the change from fornix to eyelid margin among all positions (*P* = 0.567). In group B, at all positions, the decreases from fornix to limbus or the decrease from fornix to eyelid margin were all significant (*P* = 0.000, *P* = 0.016), and there was no differences between temporal and nasal region. In group C, the changes from fornix to limbus were all significant at all positions (*P* = 0.000), and there was no differences between temporal and nasal region, the differences of change from eyelid margin to fornix was not significant (*P* = 0.376).

### Conjunctival sac volume

**Figure 2:**
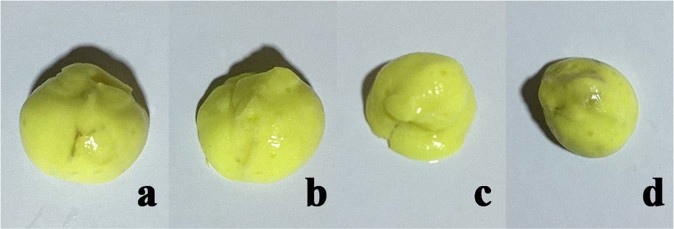
Conjunctival sac casts. (**a**) Preoperative photograph of normal conjunctival sac cast with smooth surface and natural arc-shaped margin. (**b**) Postoperative photograph from group A with surface indentation. (**c**) Postoperative photograph from group B with sulcus defect on the surface. (**d**) Postoperative photograph from group C with superior conjunctival sac absent.

The conjunctival sac casts are shown in Fig. 2. The surface of the normal conjunctival sac cast was smooth, and the margin was natural arc-shaped. The superior conjunctival sac reduced with surface indentation in group A. The superior conjunctival sac was obviously diminished with sulcus defects on the surface of the cast in group B. Most of the superior conjunctival sacs were absent on the surface of the mould in group C.

The conjunctival sac volume measurements are presented in Table 3. The mean baseline preoperative conjunctival sac volumes were 1.39 ± 0.15 cm^3^ in group A, 1.39 ± 0.16 cm^3^ in group B and 1.40 ± 0.17 cm^3^ in group C. The volumes decreased to 1.08 ± 1.29 cm^3^, 0.64 ± 0.08 cm^3^ and 0.32 ± 0.15 cm^3^, respectively, in groups A, B and C on week 4 following alkali injury. The decrease in each group was 22.03%, 54.40% and 77.64%, and the difference in the percentage decrease among the three groups was significant (*P* = 0.000).

**Table 3:** Conjunctival sac volume (cm^3^). Conjunctival sac volume decreased in groups A, B and C on week 4 following alkali injury.

Conjunctival sac volume(cm^3^)	Pre-operation	Post-operation	Δvolume	Decrement (%)	Pre VS post (*P*-value)
Group A	1.39 ± 0.15 (1.18–1.64)	1.08 ± 1.29 (0.89–1.26)	0.31 ± 0.05 (0.21–0.38)	22.03 ± 3.17 (15.41–25.41)	0.000
Group B	1.39 ± 0.16 (1.17–1.68)	0.64 ± 0.08 (0.52–0.76)	0.76 ± 0.08 (0.64–0.91)	54.40 ± 1.12 (52.73–56.02)	0.000
Group C	1.40 ± 0.17 (1.16–1.72)	0.32 ± 0.15 (0.16–0.62)	1.08 ± 0.06 (0.99–1.17)	77.64 ± 7.69 (63.62–85.97)	0.000
Decrement among groups (*P*-value)	0.000				

The difference in the percentage decrease among the three groups was significant (*P* = 0.000).

**Figure 3:**
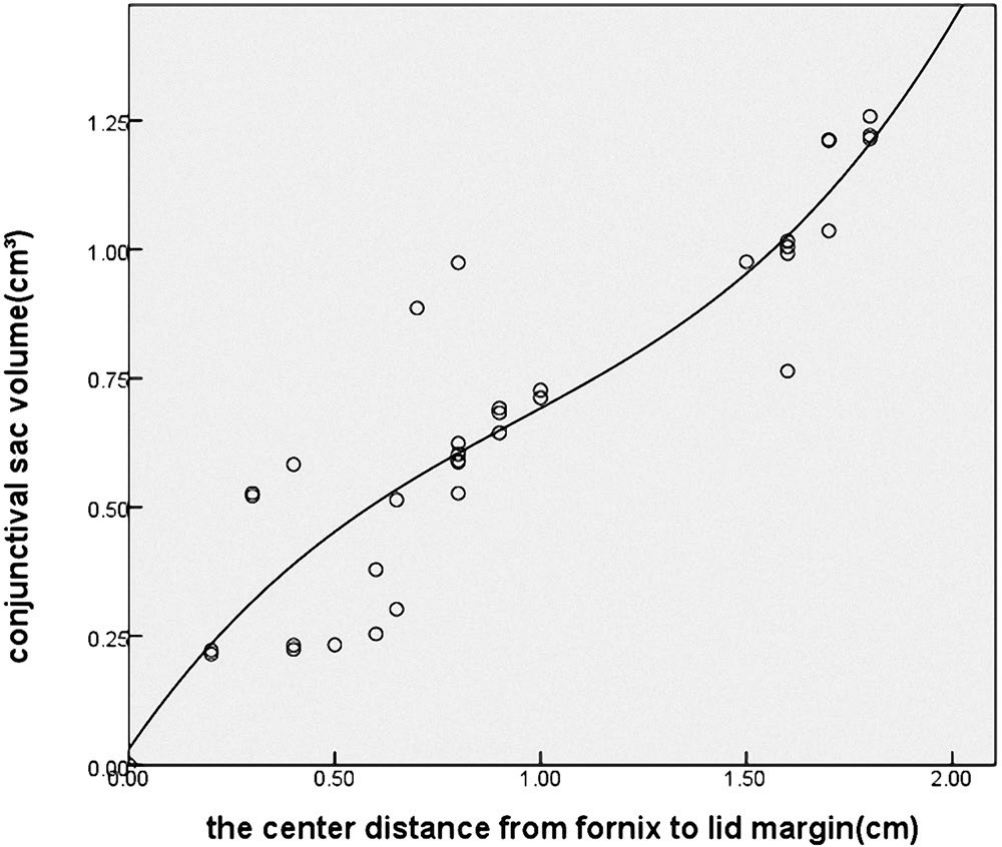
The relationship between the centre distance from fornix to lid margin and the conjunctival sac volume.

The relationship between the centre distance from the fornix to the lid margin and the conjunctival sac volume was explained by curve estimation, as shown in Fig. 3. The cubic model was significant (coefficient of determination R^2^ = 0.829, *P* = 0.000), and a curve regressive relation existed between the volume of the conjunctival sac and the length from the fornix to the lid margin.

### Severity of symblepharon

The severity of symblepharon in each group is shown in Table 4. Symblepharon severity significantly increased with the severity of alkali burns (*P* = 0.000).The proportions of grade I and grade a were 66.67% in group A, grade II (83.33%) and grade c (100%) were dominant in group b, grade III (75.00%) and grade d (66.67%) made up the majority of group C, and there was only one eye of grade IV in group C (8.33%).

**Table 4:** Grading for symblepharon severity.

Total	Group A	Group B	Group C	Symblepharon severity among groups(*P*-value)
	12	12	12	
**The centre distance from fornix to lid margin**				
I	10 (83.33%)	1 (8.33%)	0	0.000
II	2 (16.67%)	8 (66.67%)	2 (16.67%)	
III	0	3 (25.00%)	9 (75.00%)	
IV	0	0	1 (8.33%)	
**The reduction of volume**				
a	10 (83.33%)	0	0	0.000
b	2 (16.67%)	0	0	
c	0	12(100%)	4 (33.33%)	
d	0	0	8 (66.67%)	

Symblepharon severity significantly increased with the severity of alkali burns (*P* = 0.000).

### Histopathology

**Figure 4:**
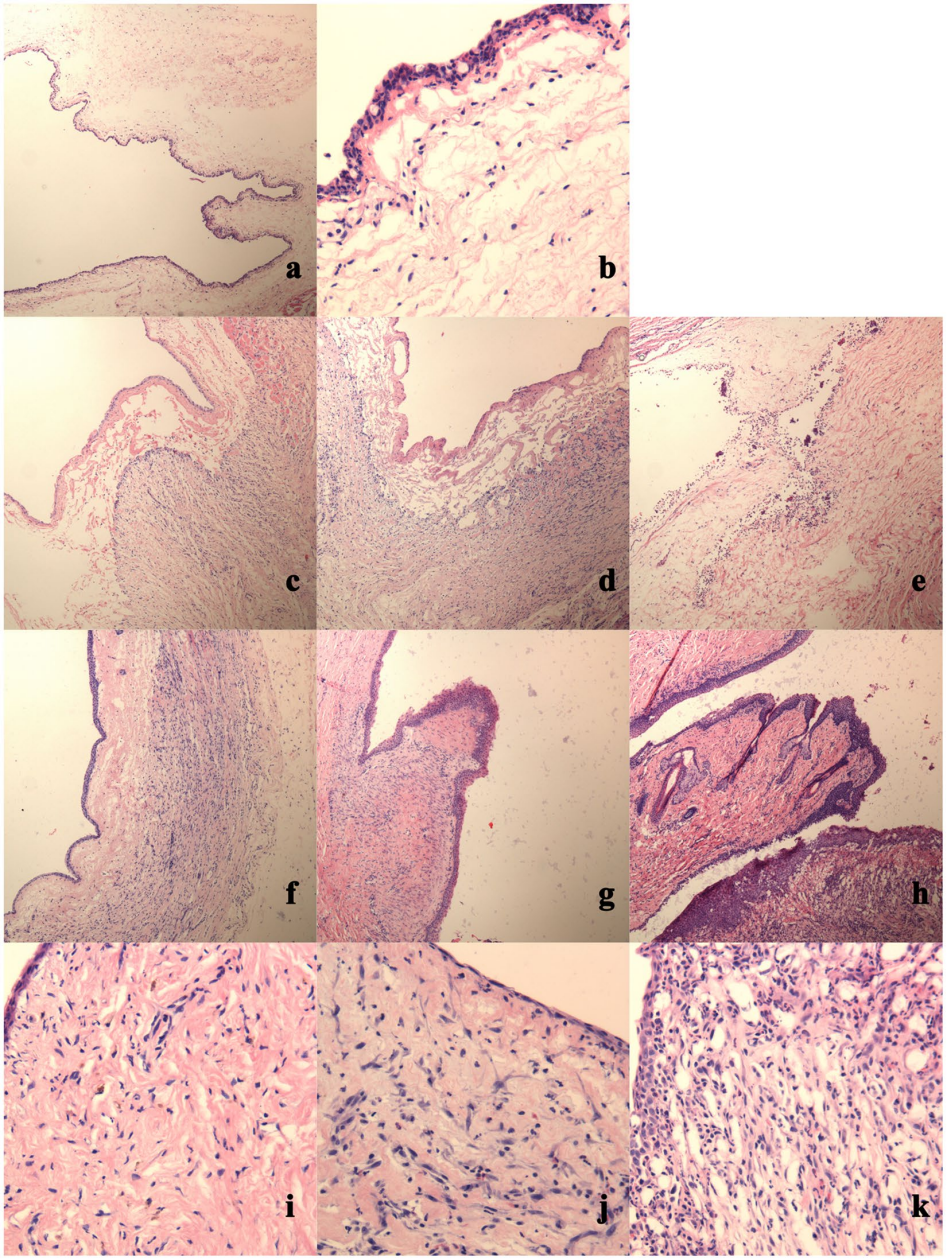
Micrographic photographs of haematoxylin-eosin staining at follow-up. (**a**,**b**) Photograph of normal conjunctiva. Conjunctiva epithelium normally consists of 2 to 3 rows of flattened surface cells and loose areolar tissue, a few goblet cells distributed in the most superficial row. (**c**–**e**) Photograph of alkali-burned superior conjunctival sac following 1 week in each group. In group A, conjunctival epithelium migration continued with the migration of fibroblasts to the burn site. In group B, re-epithelialization delayed and fibroblasts proliferated actively. In group C, necrosis was predominant. (**f**–**k**) Photograph of superior conjunctival sac on week 4 after alkali-burn procedure in the three groups. In group A, the conjunctival was normal, several mature fibroblasts lying among sub-conjunctival collagen fibres. In group B, delayed re-epithelialization, superficial band-like cicatrization and numerous juvenal fibroblasts existed. In group C, extensive conjunctival fibrosis and cicatrization (original high magnification, ×40) (original low magnification, ×200).

Micrographic photographs of haematoxylin-eosin staining at follow-up are presented in Fig. 4. The proliferation of fibrous tissue beneath the wound was observed in all groups one week post-burn, and there was extensive necrotic tissue in group C. As the damage increased, a transition phenomenon at the centre keloid tissue was observed among the three groups on the fourth week post-burn, and many fibroblasts were observed in groups A and B.

### Complications

Perforation rate: There was no corneal perforation (0%) in group A. Two eyes (16.67%) in group B had perforation of the cornea in the 3rd week, and the perforation was healed by the 4th week. In the first 2 to 3 weeks, the cornea was perforated in 9 eyes (75%) in group C, among which 6 eyes eventually healed, and 3 eyes remained with corneal ulcers. As the duration of the alkali burn increased, the corneal perforation significantly increased (*P* = 0.000).

## Discussion

The conjunctival sac (superior fornix and inferior fornix) is the blind pouch where the conjunctiva reflects upon itself between the bulbar and palpebral surfaces. The conjunctival sac depth is not clearly defined, while most researchers consider it as the depth of the conjunctival fornix, the distance from the palpebral margin to the deepest fornix. In fact, the depth of the conjunctival sac is also related to the distance from the limbus to the fornix on account of the continuity of the conjunctiva. Davis^22^ reported data of rabbit fornices and reported that the distance from the upper palpebral margin to the upper fornix was 8 mm in eye specimens. Further measurements were implemented on the superior fornix *in vivo*. For the distance from the upper palpebral margin to the upper fornix, the centre was 20.36 ± 1.20 mm, the temporal region was 19.94 ± 1.37 mm, and the nasal region was 19.03 ± 1.34 mm. These data were consistent with human studies (20 mm). For the distance from the limbus to the fornix, the centre was 13.60 ± 0. 89 mm, the temporal region was 13.10 ± 2.21 mm, and the nasal region was 12.33 ± 1.18 mm. These values were slightly more than that in humans (8–10 mm). An alginate impression material moulding was designed to show the three-dimensional morphology and the volume of the conjunctival sac. The upper eyelid was stretched to its natural maximum by subjecting to a force of 0.2 N, and the volume of the conjunctival sac was 1.39 ± 0.16 cm^3^. A cubic curve regressive relation existed between the volume of the conjunctival sac and the distance from the fornix to the lid margin.

Anatomically, when adhesion between the palpebral and bulbar conjunctiva develop and the fornix is obliterated by scar, symblepharon is formed. A good symblepharon model should meet the following requirements: a controllable burn, consistent funicular fibres, and moderate extent of fibrous adhesion without serious damage to the cornea or eyelids. NaOH solution directly dropped into the conjunctival sac or filter paper soaked in NaOH solution attached to the cornea is popular methods to induce alkali burn models. However, corneal perforation occurs frequently following extensive injury, leading to ocular atrophy. In this study, we describe improved success using a modified filter paper attached to the superior fornix. Given the forniceal depth and the contracted conjunctiva under the alkaline stimulus, the filter paper was a half-moon shape with a diameter of 10 mm, and the straight edge of the paper was 2 mm from the superior limbus and 5–6 mm from the upper eyelid margin. The filter paper soaked in NaOH solution was not as prone to complications, such as perforation or eyelid edge necrosis, as it was kept apart from the limbus and the lid margin. Eyeball rotation was controlled by anaesthetic (the eyes may be rotated to a certain degree under anaesthesia) to avoid displacement of the burn area during operation. Therefore, adhesion could form between the palpebral and the bulbar conjunctiva, moderate adhesion is beneficial for operation and observation, and corneal lesions were kept to a minimum as much as possible.

To evaluate the symblepharon severity in rabbits, a modified classification was used according to the previously reported grading system^16^. The severity was graded based on the following two parameters. The first parameter is fornix depth, defined as the centre distance from the lid margin to the deepest fornix. Kheirkhah *et al*.^16^ described the significance of fornix depth in the severity of symblepharon, and the appropriate surgical strategy could be employed for various grades. The authors disclosed that fornix reconstruction could be successfully achieved in grade II symblepharonby surgery, such as cicatrix lysis with a tissue substitute including conjunctiva, oral mucosa and amniotic membrane, or other combined additional measures. Naturally, grade II symblepharon is suitable for experimental animal studies. The second parameter is volume reduction, measured via conjunctival sac casting. Considering that most adhesion is localized, depth values may not be sufficient to present symblepharon severity. Herein, we propose a novel method for volume measurement using conjunctival sac moulding. The results showed a significant positive correlation between volume reduction and the degree of alkali burn. For adhesion was fibrous cord characterized by soft and small-scale, residual space exists around the fibrous adhesion; accordingly, ocular motility is not restricted as much. The volume actually reflects the entire conjunctival sac and can help determine the impact of adhesion on the whole conjunctival sac. This study showed a cubic curve regressive relationship between the centre distance from the fornix to the lid margin and the conjunctival sac volume, and thus, volume change was correlated with the severity of symblepharon.

Additionally, conjunctival sac casting provides a new method for evaluating conjunctival sacs. As shown in the results, the three-dimensional surface of conjunctival sac casts could be presented on the cast. The space-occupying lesion of conjunctival sacs, such as the adhesion strip, was easily identifiable on the cast. Indentations or sulcus defects were shown in groups A and B, but superior conjunctival sacs were absent in group C. Grade d (66.67%) comprised a larger portion of group C than the other two groups, although the burned area was the superior fornix. Longer durations of alkaline burn increased the permeability of the alkaline substance, causing the inferior fornix to be extensively damaged and the volume to decline sharply.

Four pathophysiologic and clinical phases have been previously introduced after chemical injury^23^, and the cornea has been more emphasized than the conjunctival sac. The analysis of rabbit fornix described herein also showed a distinct phase **(**Fig. 4**)**. In the first week, as during the early repair phase, group A was less injured, and epithelial migration continued with the migration of fibroblasts to the burn site. Group B was more injured, re-epithelialization continued and was delayed, and fibroblasts were observed. Group C involved extensive damage to the fornical conjunctival epithelium, and necrosis was predominant. After the third week, during the late repair phase, while the cornea completed phase III healing, progressive symblepharon formation was exacerbated to varying degrees. In group A, the conjunctival epithelium was clinical and phenotypical normal, several mature fibroblasts could be observed among sub-conjunctival collagen fibres, and collagen organization was improved. In group B, conjunctival epithelium re-epithelialization was delayed, superficial band-like cicatrization occurred due to an initial defect in the epithelium, and numerous juvenal fibroblasts existed in the matrix. In group C, extensive conjunctival fibrosis and cicatrization due to progressive sterile ulceration at the early stage were unusual, and ocular surface wetting abnormalities caused by abnormal mucus secretion persisted for weeks. If the epithelium continued to be absent, conjunctival erosion persisted. In brief, corresponding to the fibrovascular pannus of the cornea, fornix foreshortening or symblepharon commonly appeared at the late stage of chemical burns, the clinical course was similar to that in humans.

Nonetheless, species differences between the rabbit and human eye should be considered. Compared with humans, rabbits have much thinner tarsal border and sclera than humans^22^, which may lead to much more penetration of the alkaline substance. Rabbits have smaller exposed portion of the conjunctiva because of the obvious protrusion of the eye from the socket and the unusually wide cornea; thus, inflammatory exudates are more likely to accumulate around the wound. The fibrous tissue of the conjunctiva and the subconjunctival fascia is not as abundant in rabbit as in humans^22^, which might shrink the buffers of fibrous tissue, and the alkaline substance may diffuse across a larger area. All of these factors can affect the occurrence of symblepharon. However, the anatomical structure of the lid, including the skin, orbicularis muscle, tarsus, and conjunctiva in rabbits, is similar to that of humans^22^. For the purpose of alkali burn studies, symblepharon of the conjunctiva is similar in both species.

The present study presents a quantitative description of symblepharon, and future studies can be designed to use this quantitative system to evaluate treatments for symblepharon. For example, the studies by Khorshidi *et al*.^24^ and Horii *et al*.^25^ showed that sodium hyaluronan and thermally cross-linked gelatine film have significant effects in preventing adhesion formation. With improvements in anti-adhesion strategies, it is conceivable that alternative drugs or surgical experiments may be tailored in this animal model of symblepharon. In addition, it is important to note that, rabbits have a much smaller eyeball than humans, surgical studies may not be conducted as easy as drug experiments.

In summary, the rabbit model demonstrated stable and reliable symblepharon following alkali burn to the superior conjunctival sac. The results suggest that the duration of conjunctival sac burn is important in this rabbit model, and 90 s is a suitable duration of burn for further research. The volume measured using conjunctival sac casting should be considered when developing severity evaluation systems of symblepharon. Animal models that retain the adhesion may help further our basic understanding of symblepharon in future clinical and experimental investigations.

## Methods

### Animals

Male and female New Zealand white rabbits (supplied by the Experimental Animal Center, Chinese Academy of Medical Sciences) with no ocular disease weighing 2.0–2.5 kg and approximately 4 months of age were used in this experiment. All experiments followed the Association for Research in Vision and Ophthalmology (ARVO) Statement for the Use of Animals in Ophthalmic and Vision Research. The protocols were approved by the Animal Care Committee of Central South University (No. 2016–07–21).Animals were randomly allocated to three different groups to yield 20 animals per group. The right eyes were used for the experiment, and the left eyes were untouched. Animals were anaesthetized by intravenous injection of sodium pentobarbital (30 mg/1 mL, Merck, Darmstadt, Germany, 30 mg/kg) via the ear vein and topical application of oxybuprocaine hydrochloride eye drops (0.4%, 20 ml:80 mg, Santen Seiyaku, Osaka, Japan).

### Conjunctival sac alkali burn

To create alkali burns in group A, 10-mm-diameter Whatman filter paper was cut into halves. After soaking in 2 N of NaOH (50 μL) for 10 s, the half-moon-shaped filter papers were placed on the superior conjunctival sac for 60 s with the arced edge placed deep in the upper fornices and the straight edge placed 2 mm from the superior limbus. The conjunctival sac was then rinsed with saline until pH was approximately 7 to 7.5 (measured by pH paper). The same procedure was repeated in the other groups. The duration of filter paper contact with the conjunctival sac was 90 seconds in group B and 120 seconds in group C. After burn induction, the rabbits were observed individually in their respective cages, and levofloxacin antibiotic drops (0.5%, 1 ml:4.88 mg, Santen Seiyaku, Osaka, Japan) were applied three times daily for 4 weeks to all eyes.

### Conjunctival sac depth and conjunctival sac volume

**Figure 5:**
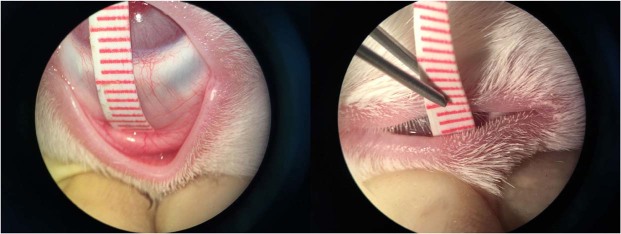
The superior conjunctival sac measurement.

The depth of the superior conjunctival sac and the volume of the conjunctival sac were measured preoperatively and on the 4th postoperative week. For the depth of the superior conjunctival sac, which conformed to the curvature of the eyeball, a plastic measuring tape with a scale starting at zero without sharp edges was inserted into the superior conjunctival sac; the limbal value was measured as the distance from the limbus to the fornix, and the eyelid margin value was measured as the distance from the deepest fornix to the lid margin **(**Fig. 5**)**. According to the quartered palpebral fissure, three locations of the superior conjunctival sac, namely, the temporal, centre and nasal locations, were measured.

For the volume of the conjunctival sac, alginate impression material moulding of the conjunctival sac was performed as follows: The eyelid was completely closed using 4–0 silk sutures (Ethicon Inc, Somerville, New Jersey, USA) at the 1/3 palpebral location and the 2/3 palpebral location through the tarsal plate of the upper and lower lid. A 2 cm loop at a distance of 1 cm from the midpoint of the upper eyelid edge was placed with a 4–0 silk suture as a traction. The upper eyelids were stretched with a force of 0.2 N by a dynamometer through the loop. One gram of alginate impression material was mixed with 7.5 mL of sterile water. When the colour of the mixture changed from orange to yellow, the emulsion was injected into the conjunctival sac through the central palpebral fissure until the emulsion overflowed. After 60 s, the sutures of the palpebral fissure were removed, and the solidified conjunctival sac impression was transferred to graduated cylinder (cylinder specification: 20 mL) pre-filled with15 mL sterile water. Recorded as the volume of the conjunctival sac, the displacement of sterile water in the graduated cylinder was suctioned and measured using a syringe (the syringe specification: 1 mL, the minimum scale: 0.01 mL). The cast models of the conjunctival sac were stored at room temperature in sterile water.

### Grading of symblepharon severity

Symblepharon severity was evaluated according to the distance from the fornix to lid margin at the middle position of the palpebral fissure and the reduction in volume (Table 5).

**Table 5:** Grading system for symblepharon severity.

Grading system for symblepharon severity	
The centre distance from fornix to lid margin	
I	≥Length of the preoperative bulbar conjunctiva
II	1/2 Length of preoperative bulbar conjunctiva <Length of palpebral conjunctiva <Length of preoperative bulbar conjunctiva
III	≤1/2 Length of preoperative bulbar conjunctiva
IV	Close to zero (ankyloblepharon)
The reduction of volume	
a	≤25%
b	25%< reduction of volume < 50%
c	50 ≤reduction of volume < 75%
d	≥75%

### Clinical observation and tissue harvest

Each right eye was examined with slit lamp microscopy and photographed.Corneal perforation was documented at 1, 2, 3 and 4 weeks following the burn.Two rabbits in each group were killed with 100 mg/kg intravenously injected pentobarbital sodium, and the eyeballs were removed for tissue section examination following haematoxylin/eosin (HE) staining at postoperative weeks 1, 2, 3 and 4.

### Statistical analysis

Statistical analysis was performed using SPSS version 18.0 (SPSS Chicago, IL).

All data are presented as the mean ± standard deviation. Fornix depth and volume were analysed using standard parametric statistics (ANOVA F-statistic). If there were differences among the three groups, Dunnett’s T3 post hoc test was used to analyse differences between two groups. The relationship between depth and volume was obtained using the curve fitting method. For comparisons of symblepharon severity, nonparametric rank statistics were used (Kruskal–Wallis H-statistic). A *P* value < 0.05 was considered significant.

## References

[1] Ang AY, Chan CC, Biber JM, Holland EJ (2013). Ocular surface stem cell transplantation rejection: incidence, characteristics, and outcomes. Cornea.

[2] Kim JY, Djalilian AR, Schwartz GS, Holland EJ (2003). Ocular surface reconstruction: limbal stem cell transplantation. Ophthalmol Clin North Am.

[3] Kaufman HE, Thomas EL (1979). Prevention and treatment of symblepharon. Am J Ophthalmol.

[4] Shi W, Wang T, Gao H, Xie L (2009). Management of severe ocular burns with symblepharon. Graefes Arch Clin Exp Ophthalmol.

[5] Azuara-Blanco A, Pillai CT, Dua HS (1999). Amniotic membrane transplantation for ocular surface reconstruction. Br J Ophthalmol.

[6] Kheirkhah A (2013). A combined approach of amniotic membrane and oral mucosa transplantation for fornix reconstruction in severe symblepharon. Cornea.

[7] Takeda K (2011). Ocular surface reconstruction using the combination of autologous cultivated oral mucosal epithelial transplantation and eyelid surgery for severe ocular surface disease. Am J Ophthalmol.

[8] Kuckelkorn R, Wenzel M, Lamprecht J, Bocking B, Reim M (1994). [Autologous transplantation of nasal mucosa after severe chemical and thermal eye burns]. Klin Monbl Augenheilkd.

[9] Choy AE, Asbell RL, Taterka HB (1977). Symblepharon repair using a silicone sheet implant. Ann Ophthalmol.

[10] Tseng SC, Di Pascuale MA, Liu DT, Gao YY, Baradaran-Rafii A (2005). Intraoperative mitomycin C and amniotic membrane transplantation for fornix reconstruction in severe cicatricial ocular surface diseases. Ophthalmology.

[11] Zhao D (2015). Sealing of the gap between the conjunctiva and tenon capsule to improve symblepharon surgery. Am J Ophthalmol.

[12] Iannetti L, Abbouda A, Fabiani C, Zito R, Campanella M (2013). Treatment of corneal neovascularization in ocular chemical injury with an off-label use of subconjunctival bevacizumab: a case report. J Med Case Rep.

[13] Fein W (1979). Repair of total and subtotal symblepharons. Ophthalmic Surg.

[14] Katircioglu YA, Budak K, Salvarli S, Duman S (2003). Amniotic membrane transplantation to reconstruct the conjunctival surface in cases of chemical burn. Jpn J Ophthalmol.

[15] Jain S, Rastogi A (2004). Evaluation of the outcome of amniotic membrane transplantation for ocular surface reconstruction in symblepharon. Eye (Lond).

[16] Kheirkhah A (2008). Surgical strategies for fornix reconstruction based on symblepharon severity. Am J Ophthalmol.

[17] Chung J, Park Y, Paek S, Chong Y, Kim W (1999). Effect of Na-hyaluronan on stromal and endothelial healing in experimental corneal alkali wounds. Ophthalmic Res.

[18] Bashkaran K (2011). Anti-inflammatory and antioxidant effects of Tualang honey in alkali injury on the eyes of rabbits: experimental animal study. BMC Complement Altern Med.

[19] Subasi S (2017). Comparison of Collagen Cross-Linking and Amniotic Membrane Transplantation in an Experimental Alkali Burn Rabbit Model. Cornea.

[20] Zhou C (2017). Sustained Subconjunctival Delivery of Infliximab Protects the Cornea and Retina Following Alkali Burn to the Eye. Invest Ophthalmol Vis Sci.

[21] Haddox JL, Pfister RR, Sommers CI, Blalock JE, Villain M (2001). Inhibitory effect of a complementary peptide on ulceration in the alkali-injured rabbit cornea. Invest Ophthalmol Vis Sci.

[22] Davis FA (1929). The Anatomy and Histology of the Eye and Orbit of the Rabbit. Trans Am Ophthalmol Soc.

[23] Wagoner MD (1997). Chemical injuries of the eye: current concepts in pathophysiology and therapy. Surv Ophthalmol.

[24] Khorshidi HR (2017). Evaluation of the effectiveness of sodium hyaluronate, sesame oil, honey, and silver nanoparticles in preventing postoperative surgical adhesion formation. An experimental study. Acta Cir Bras.

[25] Horii T (2018). Physical and biological properties of a novel anti-adhesion material made of thermally cross-linked gelatin film: Investigation of the usefulness as anti-adhesion material. J Biomed Mater Res B Appl Biomater.

